# Reduced Transferrin Levels in Active Inflammatory Bowel Disease

**DOI:** 10.1155/2017/9541370

**Published:** 2017-10-31

**Authors:** Malgorzata Matusiewicz, Katarzyna Neubauer, Paulina Lewandowska, Andrzej Gamian, Malgorzata Krzystek-Korpacka

**Affiliations:** ^1^Department of Medical Biochemistry, Wroclaw Medical University, Chalubinskiego 10, 50-368 Wroclaw, Poland; ^2^Department of Gastroenterology and Hepatology, Wroclaw Medical University, Borowska 213, 50-556 Wroclaw, Poland; ^3^Ludwik Hirszfeld Institute of Immunology and Experimental Therapy, Polish Academy of Sciences, Weigla 12, 53-114 Wroclaw, Poland

## Abstract

Inflammatory bowel disease (IBD) is an inflammatory disease of unclear etiopathogenesis and challenging diagnosis, frequently complicated by anemia and malnutrition. C-reactive protein (CRP) remains the only biochemical marker of clinical relevance. The aim of this study was to test hypothesis that transferrin, coinfluenced by inflammation, malnutrition, anemia, and oxidative stress, may better reflect global IBD patient's condition than any other more specific index. Transferrin and other indices of inflammation, anemia, malnutrition, and oxidative stress were measured in 137 IBD patients (Crohn's disease (CD): *n* = 63 and ulcerative colitis (UC): *n* = 74) and 97 controls. Transferrin is reduced in active CD and UC and negatively correlates with the disease activity scores (CD: *ρ* = −0.49; UC: *ρ* = −0.52). In UC, transferrin correlates negatively with CRP, erythrocyte sedimentation rate (ESR), leukocytes, platelets, interleukin-6, interleukin-10, and TNF-*α* and positively with albumins, cholesterol, hemoglobin, hematocrit, erythrocytes, iron, and paraoxonase-1. In CD, transferrin correlates negatively with CRP, leukocytes, platelets, interleukin-1, and interleukin-6 and positively with albumins, iron, catalase, glutathione peroxidase-1, superoxide dismutase-1, and paraoxonase-1. The associations with inflammation and anemia/malnutrition were more pronounced in UC and with oxidative stress in CD. As UC activity marker, transferrin outperforms ESR and hemoglobin, indices used in calculating the disease clinical severity score.

## 1. Introduction

Inflammatory bowel disease (IBD) is a debilitating, chronic, relapsing, and currently incurable inflammatory disease, the two major forms of which are Crohn's disease (CD) and ulcerative colitis (UC). The etiopathogenesis of IBD remains unclear with the combined effect of genetic, immunological, and microbiological factors [1, 2]. Recently, the contribution of oxidative stress in the disease initiation and progression has been suggested [3, 4]. Despite intensive studies also a question of biomarkers which would aid in diagnosis, differentiation, and monitoring of the disease course and treatment mode remains open. In recent years, this problem has gained new interest, especially that there is often a poor correlation between clinical symptoms and endoscopic findings [5, 6]. Therefore, it is not surprising that every year new biomarkers are being proposed. Some of them, such as faecal calprotectin, have already found their application in IBD diagnostics and some, such as calgranulin C and B Cell-Activating Factor (BAFF), show potential as new diagnostic tool [7–9]. Still, a pursuit of new reliable, noninvasive, easy to perform and nonexpensive biomarkers is one of the main goals of IBD connected research [5].

Anemia is a common extraintestinal manifestation of IBD affecting up to 75% of patients [10, 11]. Its pathogenesis is multifactorial, encompassing iron deficiency anemia (IDA) and anemia of chronic disease (ACD), conditions frequently overlapping, with marginal contribution of folic acid and vitamin B_12_ deficiencies [12] or drug-induced anemia [11, 13, 14]. IDA in IBD results from chronic blood loss, reduced iron intake, and decreased uptake from enterocytes, while ACD is associated with inflammation and upregulated hepcidin expression, mainly by IL-6, shortened red blood cells lifespan, and hampered bone marrow response to erythropoietin [10, 11, 15]. Protein-energy malnutrition (PEM) is yet another common systemic manifestation of IBD with prevalence rates reaching those of anemia and similarly complex etiology [16, 17]. It encompasses reduced food intake, impaired digestion, and absorption, accompanied by enhanced protein loss in the course of systemic inflammation and treatment with steroids [16].

Transferrin is a glycoprotein capable of reversible iron binding, which serves as its transporter between iron donating enterocytes and macrophages and accepting erythroblasts. Systemic levels of transferrin differently respond to anemia depending on its cause, being elevated in IDA but normal/decreased in ACD. However, its utility as a differentiating marker in IBD-associated anemia is questioned since transferrin concentration varies also in response to inflammation and malnutrition [10].

The aim of our study was to evaluate transferrin levels in a large cohort of IBD patients and relate it to indices of inflammation, malnutrition, anemia, and oxidative stress. We hypothesized that a lack of specificity caused by its relation to the above-mentioned IBD-accompanying conditions might be advantageous and transferrin may better reflect the overall disease severity than any other more specific index.

## 2. Materials and Methods

### 2.1. Study Population

A cohort of 234 individuals was examined: 97 apparently healthy controls (24 volunteers recruited from hospital staff and university students and 73 blood donors from the Regional Center for Blood Donation and Therapeutics in Wroclaw, Poland) and 137 IBD patients: 63 with Crohn's disease (36 with active and 27 with inactive disease) and 74 with ulcerative colitis (28 with active and 46 with inactive disease). IBD patients were recruited from among patients admitted to the Department of Gastroenterology and Hepatology of Wroclaw Medical University and First Department and Clinic of General, Gastroenterological and Endocrinological Surgery of Wroclaw Medical University. Patients with the coexistence of other severe systemic diseases, malignancies, or pregnancies were not included. Crohn's disease activity was assessed using Crohn's Disease Activity Index (CDAI) combining the evaluation of vital parameters, clinical findings, and medical history as described in detail elsewhere [18]. For the assessment of UC activity, we applied Rachmilewitz Activity Index (RI; ranging from 0 to 23), which encompasses stool frequency, number of stools with blood, general well-being, abdominal pain/cramp, fever, extraintestinal manifestations, and laboratory tests: erythrocyte sedimentation rate and hemoglobin concentration. Endoscopic findings in UC patients were evaluated as follows: a score of 0 was allocated for inactive disease, 1 for mild disease (erythema, decreased vascular pattern, and mild friability), 2 for moderate disease (marked erythema, lack of vascular pattern, friability, and erosions), and 3 for severe disease (spontaneous bleeding, ulceration). Almost all patients were treated with 5′-ASA derivatives. 19 CD and 24 UC patients were treated with corticosteroids; 18 CD and 18 UC patients were given azathioprine. Anemia was defined as a drop in hemoglobin level below 12 g/dL in female and 13 g/dL in male patients. There were 27 CD patients (7 in remission) and 24 UC patients (9 in remission) with anemia.

Characteristics of study population are depicted in Table 1.

The study protocol was approved by the medical ethics committee of our university and was in accordance with the ethical standards formulated in the Helsinki Declaration of 1975. Informed consent was obtained from all of the subjects.

### 2.2. Analytical Methods

Blood drawn by venous puncture in a fasting state was collected into serum-separator tubes, clotted, and centrifuged (30′, 720 ×g). The obtained serum was stored at −80°C until examination. All parameters were measured in series as single day experiments to assure uniform assay conditions. Prolonged storage was avoided.

Transferrin was assessed using the enhanced immunoturbidimetric method (intra- and interassay CVs for this assay were <2%) and albumin with bromocresol blue method using appropriate kits provided by Stamar (Dabrowa Gornicza, Poland) in accordance with the supplier's protocols. Immunoturbidimetry has been found the most accurate method of transferrin determination [19]. Interleukins-1, -6, and -10 (IL-1, -6, and -10) and tumor necrosis factor- (TNF-) *α* were measured by an enzyme double-antibody indirect immunoassays with PeliKine Compact human IL-1, IL-6, IL-10, and TNF-*α* ELISA kits, respectively, supplied by Sanquin (Amsterdam, Holland). IL-1, IL-6, and TNF-*α* were measured in 42 CD patients and 51 UC patients. Iron was measured colorimetrically using chromazurol B method in a presence of lipid clearing factor (LCF) using an iron assay kit provided by Emapol (Gdansk, Poland). Other laboratory indices were assessed with routine automatic procedures. Data on enzymatic antioxidants in examined cohort of IBD patients were retrieved from our earlier papers [20, 21] for a purpose of correlation analysis; in case of paraoxonase-1, the enzyme activity was measured as its arylesterase activity and data were available for 43 CD and 52 UC patients.

### 2.3. Statistical Analysis

Data distribution was examined using Kolmogorov-Smirnov normality test. Data on transferrin were normally distributed and hence are presented as means ± SD; the remaining data are presented either as means ± SD or as medians with IQR. Normally distributed data are analyzed using* t*-test for independent samples or 1-way ANOVA, while nonnormally distributed data are analyzed using Mann–Whitney *U* test or Kruskal-Wallis *H* test. Correlation analysis was conducted using Pearson correlation test (*r*) or Spearman correlation test (*ρ*) depending on data distribution and character. Frequency analysis was conducted using *χ*^2^ test. Multiple regression (stepwise method) was used to coexamine transferrin and other parameters as predictors of CD and UC severity and evaluate the strength of identified associations. Receiver Operating Characteristics (ROC) curve analysis was employed to evaluate its diagnostic value. Marker overall diagnostic accuracy was expressed in terms of the area under the ROC curve (AUC) with 95% CI and *p* for the difference between the calculated AUC and AUC = 0.5 (index with no discriminating power). Sensitivities and specificities matching a cut-off value corresponding to the highest accuracy (minimal false positives and false negatives) and Youden's index combining sensitivity and specificity (sensitivity (expressed as a part of a whole number) + specificity − 1) were determined as well.

All calculated *p* values were two-sided and *p* ≤ 0.05 was considered significant. The entire statistical analysis was conducted with MedCalc Statistical Software version 17.2 (MedCalc Software bvba, Ostend, Belgium; https://www.medcalc.org; 2017).

## 3. Results

**Figure 1:**
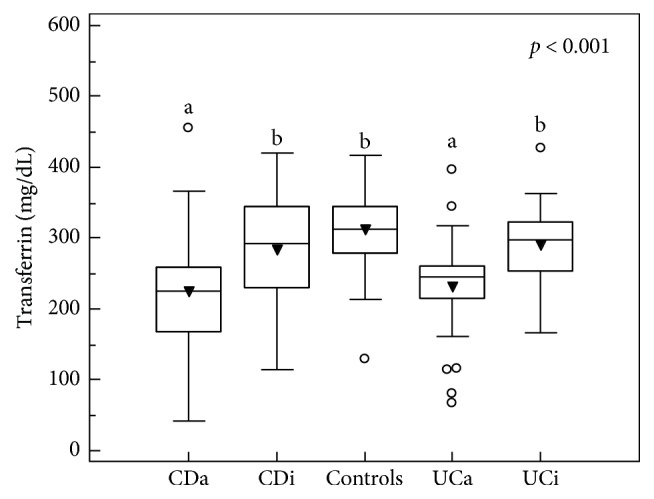
Transferrin levels in IBD patients as compared to healthy volunteers. CDa: active Crohn's disease; CDi: inactive Crohn's disease; UCa: active ulcerative colitis; UCi: inactive ulcerative colitis; ^a^significantly different from controls and inactive CD and UC; ^b^significantly different from active CD and UC; triangles represent mean values, boxes represent median values with interquartile range and whiskers represent minimum and maximum excluding outside values (open circles).

**Figure 2:**
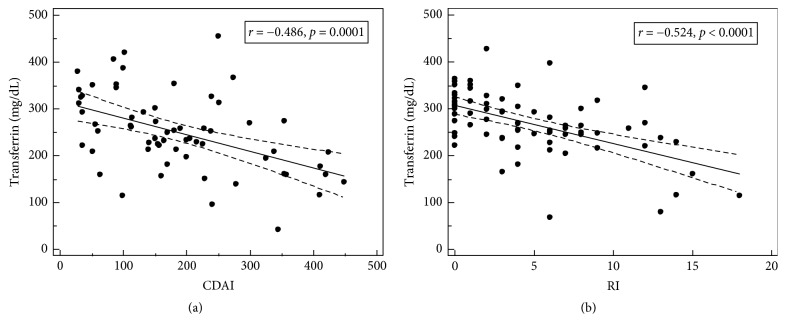
Correlation between transferrin levels and (a) Crohn's Disease Activity Index (CDAI) and (b) Rachmilewitz index (RI; ulcerative colitis activity index). Regression line is depicted as a straight, solid line accompanied by 95% CI represented by dotted lines.

We observed a significant decrease in transferrin levels in patients with active CD and UC, without differences between these two IBD forms (Figure 1). Transferrin levels negatively correlated with indices of disease clinical activity: CDAI in CD patients (Figure 2(a)) and RI in UC patients (Figure 2(b)), in whom it also correlated with endoscopic score (*ρ* = −0.373, *p* = 0.001).

Transferrin in IBD patients negatively correlated with inflammatory indices, with the strongest correlations with IL-6 and hsCRP in CD and IL-6, WBC, and TNF-*α* in UC, although the latter association was found exclusively in patients with active disease (Table 2).

We examined transferrin association with indices of nutritional status and anemia. We found transferrin to positively correlate with all examined indices (albumin, cholesterol, hemoglobin, hematocrit, RBC, and iron) in UC patients but only with albumin and iron in CD patients (Table 3).

We also examined the associations with antioxidants: serum paraoxonase-1 and erythrocyte catalase, glutathione peroxidase-1, and superoxide dismutase-1. Transferrin positively correlated with all of these parameters in CD patients but in case of UC patients exclusively with paraoxonase-1 (Table 4).

For comparative purposes, we examined correlation of other inflammatory indices with CDAI and RI. hsCRP, ESR, PLT, and IL-6 positively correlated with both CDAI and RI, while WBC only correlated with CDAI. No significant correlations were found for IL-1, IL-10, and TNF-*α*. From among antioxidants, glutathione peroxidase-1 and paraoxonase-1 correlated with CDAI and RI, while superoxide dismutase-1 correlated with CDAI. No significant correlations were found for catalase. Iron, HGB, and albumins correlated with both CDAI and RI, while cholesterol exclusively correlated with RI (Table 5).

To evaluate whether transferrin correlation with CDAI or RI truly reflects its association with the disease severity and is not mediated by transferrin association with inflammatory, nutritional, and anemia indices or antioxidants, we employed multiple regression. Paired transferrin and other indices were successively tested as independent variables explaining variability in CDAI or RI in CD and UC, respectively. In UC, transferrin was always retained in the regression model. It was an exclusive RI predictor when evaluated together with IL-6, CRP, albumins, cholesterol, and MCV, which explained 30% in RI variability. In other models, transferrin and ESR, PLT, HGB, paraoxonase-1, or iron were independently associated with RI. Together with platelets, transferrin explained 49% in RI variability. The results obtained in CD were similar with the exception of transferrin-albumin and transferrin-CRP models, in which albumin and CRP but not transferrin were independent predictors of CDAI and transferrin-paraoxonase-1 model, in which exclusively transferrin was independently associated with CDAI. Transferrin occurred to mediate superoxide dismutase-1 association with CDAI but not that of glutathione peroxidase-1. Transferrin alone explained 23.5% in CDAI variability and 46% together with hemoglobin or platelets.

**Figure 3:**
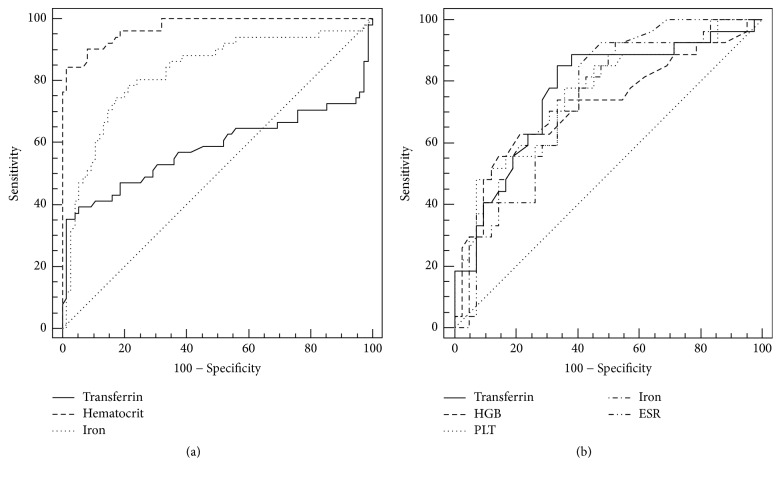
Transferrin performance (a) as an anemia indicator in IBD and (b) as a marker of the disease activity.

Transferrin only tended to be decreased in anemic patients: 245 (216–274) versus 271 (259–282) mg/dL, *p* = 0.106 and its diagnostic accuracy defined as the area under ROC curve was bad: AUC = 0.58 (0.49–0.67), *p* = 0.189. Although transferrin had high specificity (99% at ≤182 mg/dL), its sensitivity was low (35%) and transferrin was significantly outperformed by iron (resp., AUC = 0.83 (0.75–0.89), *p* < 0.0001; 82 and 75% at ≤11.26 *μ*M) and HCT (resp., AUC = 0.97 (0.93–0.99), *p* < 0.0001; 99 and 84% at ≤35.8) (Figure 3(a)).

The performance of transferrin as a marker of active UC was good with AUC = 0.763 (0.650–0.854), *p* < 0.0001, and sensitivity and specificity at ≤279 mg/dL were 86 and 65%. Comparably good was the performance of ESR (AUC = 0.767, 85, and 60% at >12 mm/h), PLT (AUC = 0.761, 78, and 65% at >293 × 10^9^/L), iron (AUC = 0.743, 93, and 49% at ≤17.6 *μ*M), HGB (AUC = 0.720, 63, and 79% at <12.2 g/dL), and paraoxonase-1 (AUC = 0.749, 75 and 78% at ≤132.5 U) (Figure 3(b)). Youden's index for transferrin was the highest (0.51) and these of other indices were all below 0.5 (resp., 0.45, 0.43, 0.42, 0.43, and 0.42 for ESR, PLT, iron, HGB, and paraoxonase-1).

## 4. Discussion

Oxidative stress, inflammation, anemia, and malnutrition often coexist in active IBD, inciting each other. Inflammation triggers ACD as proinflammatory cytokines interfere with iron homeostasis and contributes to malnutrition by affecting muscle protein turnover. Undernutrition, in turn, may lead to anemia due to iron, foliate, and/or vitamin B_12_ deficiencies. Oxidative stress is exacerbating (if not inducing) inflammation by attracting neutrophils and macrophages into damaged tissue, which respond by releasing reactive oxygen species (ROS), deepening oxidants/antioxidants imbalance. Moreover, ROS are implicated in induction of muscle protein degradation [22], while malnutrition-associated hypoalbuminemia diminishes antioxidant capacity of serum. Nowadays, a great deal of effort is undertaken to identify specific markers that may help to detect and evaluate the severity of each of these conditions separately and thus facilitating administration of appropriate treatment. Going against the tide, however, we wondered whether a less specific marker, the levels of which vary in relation to all these conditions, may better assess global patient's condition and could be useful in monitoring of IBD patients. Such a biomarker might meet the goals set by the Precision Medicine Initiative, which identified biomarkers studies as one of the key scientific opportunities [5]. We examined transferrin, an index coinfluenced by oxidative stress and inflammatory, nutritional, and iron status and, hence, thought to be too nonspecific to be of use in complex diseases like IBD.

Transferrin is an iron deficiency marker, superior to saturation-based indices and iron levels [23]. It also owns the potential as a differentiating tool: the levels of transferrin differently respond to IDA and ACD being elevated in the former and normal/decreased in the latter [10]. Nonetheless, transferrin measurement is recommended only in an extensive workup for anemia in IBD patients [24]. Accordingly, we found transferrin to be significantly decreased in both CD and UC, corresponding with the presentation of mixed-type anemia [10]. It positively correlated with indices of anemia, particularly iron, more pronouncedly in UC than CD. It has to be noted that Alves et al. reported that anemia was associated with clinical disease activity index only in case of UC and not CD [25]. However, as a marker of anemia, defined by a drop in hemoglobin concentration, transferrin overall performance was bad. It only tended to be decreased in anemic patients, a probable effect of opposite impact of IDA and ACD. In a subgroup of anemic patients in remission, hence without ACD interference, transferrin tended to be elevated (data not shown), consistently with observations on persisting iron and vitamin deficiencies in inactive IBD [26]. Transferrin is also considered to be a reliable measure of overall protein status. However, of the biochemical markers, albumin is recommended as PEM marker in active IBD patients [16]. Correspondingly, we found transferrin to positively correlate with albumin as well as cholesterol, a less specific index of poor nutritional status. Again, the association was more pronounced in UC. Transferrin is a negative acute phase reactant as well and as such decreases in response to inflammation. Yet, it is CRP that is universally measured to indicate ongoing inflammation and evaluate its severity. Accordingly, transferrin correlated with all measured inflammatory indices and, except for hsCRP and IL-1, these associations were stronger in UC than CD. By sequestering iron, transferrin prevents its involvement in free radical production in the Haber-Weiss reaction, constituting an important component of serum antioxidant defense. In agreement with this fact, transferrin was associated with the activities of enzymatic antioxidants: serum paraoxonase-1, erythrocyte superoxide dismutase-1, catalase, and glutathione peroxidase, yet, except for paraoxonase-1, exclusively in CD.

A decrease in transferrin concentrations occurred solely in active IBD. This might corroborate the findings that anti-TNF therapy has resulted in the improvement of iron availability and in the increase in transferrin concentrations [27, 28]. Interestingly, we found some differences between CD and UC concerning transferrin; it was generally more closely associated with oxidative stress and more loosely with inflammation (except for hsCRP and IL-1) and anemia/nutritional status in CD. Consequently, transferrin better corresponded with RI than CDAI. As hypothesized, the marker's lack of specificity occurred to be advantageous since transferrin displayed the highest correlation coefficients with clinical assessment of disease severity in UC. As a more general severity marker, transferrin outperformed hemoglobin and ESR, parameters used in RI calculation. Transferrin alone explained almost one-third of variability in RI. Moreover, it better explained the variation in RI than hsCRP, the key inflammatory index, or IL-6, a multifunctional proinflammatory cytokine involved in ACD development [29] and muscle wasting [22]. It mediated albumin, cholesterol, and MCV association with RI as well. Together with platelets, transferrin explained half of variability in RI. We examined transferrin performance as an active UC indicator by ROC analysis and found it to display a good diagnostic accuracy, similar to that of hemoglobin, iron, ESR, platelets, and paraoxonase-1 with, however, superior combined sensitivity and specificity. The fact that transferrin was associated with RI independently from the other markers raises possibility that their coevaluation might be advantageous. However, transferrin performance needs to be reevaluated on a larger population. Since transferrin occurred to be a promising marker in UC only, the ROC analysis was restricted to UC patients (active versus inactive) what limited the number of observations and might weaken the power of analysis. Nevertheless, our results merit further studies evaluating transferrin as a prognostic factor in IBD, particularly in UC.

## 5. Conclusions

Reduction in transferrin levels reflects IBD activity and severity of inflammation, more tightly in UC. Transferrin is associated with anemia/malnutrition in UC while with oxidative stress in CD. As UC activity marker, transferrin outperforms ESR and hemoglobin, indices used in calculating the disease clinical severity score.

## Figures and Tables

**Table 1:** Characteristics of study population.

	Controls	Crohn's disease		Ulcerative colitis		*p* value
		Active	Inactive	Active	Inactive	
Sex, F/M	40/57	19/17	14/13	14/14	20/26	0.696
Age, yrs. (range)	37.3 (21–63)	36.8 (20–68)	35.2 (19–69)	39.5 (19–79)	42.2 (18–74)	0.165
Age at onset, yrs.	—	34.3 ± 14	34 ± 11	34.8 ± 15	39.9 ± 15	0.282
Duration, yrs.	—	6.6 ± 6.7	4.4 ± 5.3	7.6 ± 9.1	9.7 ± 8.3	0.076
Activity (CDAI or RI)	—	252.8 ± 90	97.7 ± 79	9.1 ± 3.7	2.0 ± 3.2	—
HGB, g/dL	—	11.7 ± 1.9	12.8 ± 1.5	11.7 ± 1.9	13.1 ± 1.4	0.001
HCT, %	—	35.9 ± 5.1	38.9 ± 4.4	35.4 ± 5.7	39.7 ± 3.6	0.001
RBC, ×10^12^/L	—	4.41 ± 0.7	4.66 ± 0.4	4.18 ± 0.6	4.61 ± 0.45	0.010
Iron, *µ*M	19.7 ± 8	10.4 ± 6.6	15.9 ± 6.5	11 ± 5.5	16.5 ± 7.5	<0.001
Albumin, g/dL	4.74 ± 0.37	4.06 ± 0.65	4.53 ± 0.48	4.17 ± 0.62	4.59 ± 0.40	<0.001
hsCRP, mg/L	2.6 ± 5.4	41 ± 48	13.1 ± 34	23.2 ± 29	18 ± 58	0.001
ESR, mm/h	—	37 ± 25	18 ± 18	34 ± 21	18 ± 18	<0.001
WBC, ×10^9^/L	—	7.37 ± 2.7	6.61 ± 3.7	8.89 ± 4.2	7.11 ± 2.7	0.086
PLT, ×10^9^/L	—	422 ± 153	277 ± 113	389 ± 138	278 ± 74	<0.001

If not otherwise stated, data presented as means ± SD; F/M: female to male ratio; CDAI: Crohn's Disease Activity Index; RI: Rachmilewitz index; HGB: hemoglobin; HCT: hematocrit; RBC: red blood cells; hsCRP: high sensitive CRP; ESR: erythrocyte sedimentation rate; WBC: white blood cells; PLT: platelets.

**Table 2:** Correlation between transferrin levels in IBD patients and inflammatory indices.

	Crohn's disease	Ulcerative colitis
hsCRP	*ρ* = −0.521, *p* = 0.0001	*ρ* = −0.293, *p* = 0.022
ESR	NS	*ρ* = −0.314, *p* = 0.008
WBC	*r* = −0.322, *p* = 0.016	*r* = −0.454, *p* = 0.0001
PLT	*r* = −0.277, *p* = 0.044	*r* = −0.347, *p* = 0.003
IL-1	*ρ* = −0.329, *p* = 0.036	NS
IL-6	*r* = −0.465, *p* = 0.002	*r* = −0.485, *p* = 0.0003
IL-10	NS	*ρ* = −0.361, *p* = 0.050^*∗*^
TNF-*α*	NS	*ρ* = −0.571, *p* = 0.009^*∗*^

^*∗*^Exclusively in active disease; NS: not significant; *r*: Pearson correlation coefficient; *ρ*: Spearman correlation coefficient; hsCRP: high sensitive CRP; ESR: erythrocyte sedimentation rate; WBC: white blood cells; PLT: platelets; IL: interleukin; TNF-*α*: tumor necrosis factor-*α*.

**Table 3:** Correlation between transferrin levels in IBD patients and nutritional indices.

	Crohn's disease	Ulcerative colitis
Albumins	*r* = 0.582, *p* < 0.0001	*r* = 0.668, *p* < 0.0001
Cholesterol	NS	*r* = 0.431, *p* = 0.0005
HGB	NS	*r* = 0.236, *p* = 0.049
HCT	*r* = 0.260, *p* = 0.055	*r* = 0.281, *p* = 0.018
RBC	NS	*r* = 0.261, *p* = 0.029
Iron	*r* = 0.284, *p* = 0.025	*r* = 0.312, *p* = 0.007^*∗*^

NS: not significant; *r*: Pearson correlation coefficient; HGB: hemoglobin; HCT: hematocrit; RBC: red blood cells; ^*∗*^*r* = 0.38, *p* = 0.047 in active disease.

**Table 4:** Correlation between transferrin levels in IBD patients and antioxidants.

	Crohn's disease	Ulcerative colitis
Catalase	*r* = 0.536, *p* = 0.002^*∗*^	NS
GPx1	*r* = 0.320, *p* = 0.022	NS
SOD1	*r* = 0.375, *p* = 0.037^*∗*^	NS
PON1	*r* = 0.461, *p* = 0.002	*r* = 0.537, *p* < 0.0001

^*∗*^Exclusively in active disease; NS: not significant; *r*: Pearson correlation coefficient; GPx-1: glutathione peroxidase-1; SOD1: superoxide dismutase-1; PON1: paraoxonase-1.

**Table 5:** Correlation between IBD severity and inflammatory, nutritional, anemia, and oxidative stress indices.

	Crohn's disease	Ulcerative colitis
hsCRP	*ρ* = 0.64, *p* < 0.001	*ρ* = 0.36, *p* = 0.004
ESR	*ρ* = 0.528, *p* < 0.001	*ρ* = 0.469, *p* < 0.001
PLT	*ρ* = 0.598, *p* < 0.001	*ρ* = 0.409, *p* < 0.001
IL-6	*ρ* = 0.669, *p* < 0.001	*ρ* = 0.490, *p* < 0.001
WBC	*ρ* = 0.269, *p* = 0.047	NS
HGB	*ρ* = −0.567, *p* < 0.001	*ρ* = −0.416, *p* < 0.001
Iron	*ρ* = −0.535, *p* < 0.001	*ρ* = −0.443, *p* < 0.001
MCV	NS	*ρ* = −0.25, *p* = 0.037
Albumins	*ρ* = −0.494, *p* < 0.001	*ρ* = −0.436, *p* < 0.001
Cholesterol	NS	*ρ* = −0.31, *p* = 0.014
GPx1	*ρ* = −0.453, *p* < 0.001	*ρ* = −0.278, *p* = 0.026
SOD1	*ρ* = −0.289, *p* = 0.036	NS
PON1	*ρ* = −0.449, *p* = 0.003	*ρ* = −0.487, *p* < 0.001

hsCRP: high sensitive CRP; ESR: erythrocyte sedimentation rate; PLT: platelets count; WBC: leukocyte count; HGB: hemoglobin; MCV: mean corpuscular volume; GPx1: glutathione peroxidase-1; SOD1: superoxide dismutase-1; PON1: paraoxonase-1; NS: not significant.
